# Decoding the space integrome: Personalized countermeasures for a mission to Mars

**DOI:** 10.1113/EP092629

**Published:** 2025-02-21

**Authors:** Damian M. Bailey

**Affiliations:** ^1^ Neurovascular Research Laboratory, Faculty of Life Sciences and Education University of South Wales Pontypridd UK

**Keywords:** astronaut, integrative physiology, Mars, space exposome, space integrome

1

Only those who will risk going too far can possibly find out how far it is possible to go.

T. S. Eliot (1888–1965)

2

On 14 January 2004, the USA set forth a ‘Vision for Space Exploration’, outlining ambitious plans to establish human research stations and habitats with rotating crew on the Moon and, eventually, Mars. With the decommissioning of the International Space Station (ISS) planned for ∼2030, the Artemis programme is now set to make this dream a reality, with a crewed mission to Mars extending beyond 1000 days, scheduled as early as the late 2040s. This exciting era in deep space exploration will face unique challenges as humankind looks to transform from a terrestrial to extraterrestrial species, placing unprecedented demands on an astronaut's health, performance and medical needs.

Indeed, there is no environment more unique or challenging to humans than outer space. Astronauts will have to face the full force of the space ‘exposome’, an emergent concept originally founded in toxicology (Bliss, 1939), which reflects the cumulative sum of all environmental hazards encountered during spaceflight: cosmic radiation, isolation and confinement, distance from Earth, hostile/closed environments and altered gravity fields (Figure 1a; Patel et al., 2020). According to NASA's Human Research Program (NASA, 2023), these hazards are associated with >30 human health risks, with several classified as high priority or ‘red’ rated owing to their likelihood and consequence, across the temporal continuum of acute in‐mission to those emerging in later life (Romero & Francisco, 2020). Addressing these hazards, defining risks and providing solutions remains a top priority for space agencies and commercial partners alike, occupying the best scientific minds of the 21st century.

**FIGURE 1:**
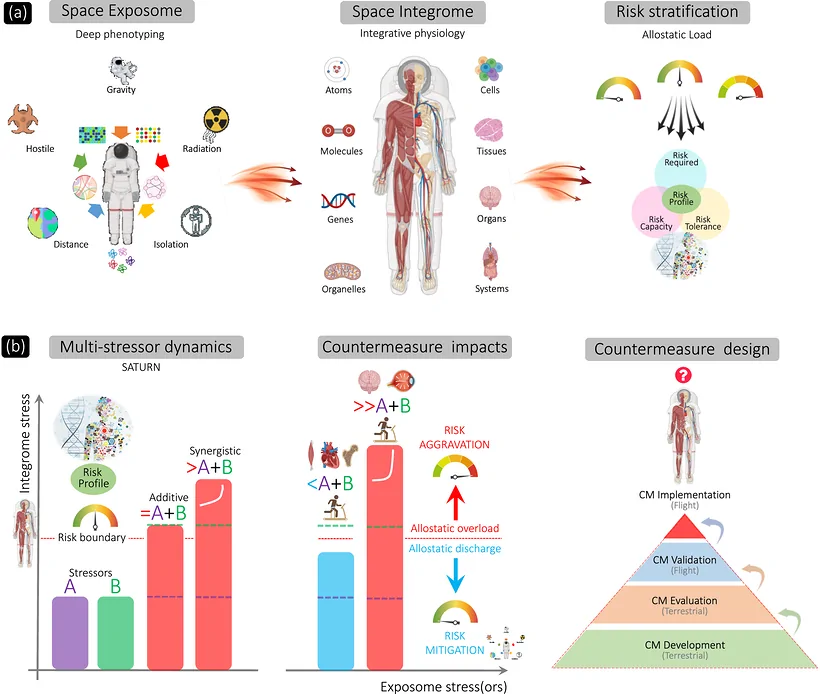
Characterizing (a) and containing (b) the space integrome. (a) The space exposome reflects the totality or cumulative sum of all environmental exposures an astronaut experiences during spaceflight, whereas the integrome reflects the integrative physiological responses, including unforeseen connections, that impact allostatic load [herein defined as the cumulative physiologial burden (McEwen, 1998)] and challenge to systemic homeostasis. There is a strategic need to move beyond traditional univariate approaches constrained to how a single organ system responds to the space exposome to a deeper phenotyping of the multivariate integrative responses to optimize risk stratification and corresponding mitigation, herein defined as the space integrome. (b) The systemic response is highly complex and difficult to model given the synergies between environmental stressors, multitude of organ systems affected and extraneous factors. Multiple stressors (SpAce inTegrome mUlti‐stRessor dyNamics, acronymized to SATURN) can impact integrome stress independently (linear additive: = A + B) or interact to compound stress in a complex, non‐linear and unpredictable manner (synergistic: > A + B) (Pirotta et al., 2022). Although a countermeasure (CM) is designed to mitigate risk by reducing the allostatic load to an ‘acceptable’ (albeit undefined) level (antagonistic: < A + B), this is highly organ specific, and one size does not fit all. For example, exercise CMs have been shown to mitigate (allostatic discharge) spaceflight‐induced musculoskeletal, cardiac and bone degeneration, whereas they might aggravate (allostatic overload) neurological complications (see main text). This poses serious challenges for the design and ultimate implementation of optimized systemic CMs.

In this issue of *Experimental Physiology*, Fernandez‐Gonzalo et al. (2026) focus on the application of one such solution, exercise countermeasures (CMs), which are targeted interventions designed to mitigate risk. Spaceflight CMs have enjoyed a rich history dating back to the early 1950s, prior to formal implementation of the first exercise CMs for the US Gemini program (Scott et al., 2019). In their elegant review, the authors highlight the need to address critical knowledge gaps that have traditionally been ignored by the extant literature that, if addressed, could improve CM efficacy and corresponding risk mitigation (Fernandez‐Gonzalo et al., 2026). These include focused consideration of exercise CMs that: (1) optimize the systemic (i.e. multi‐organ) responses, emphasizing the need for more multidisciplinary research; (2) are tailored and reflect individualized training plans to enhance enjoyment and compliance; (3) address time‐dependent load variabilities to better determine the ‘minimal effective dose’ required to preserve physiological function in a pragmatic manner; (4) take into account altered nutrition (i.e. hypocaloric diets); and (5) are based on sound experimental design principles that mandate inclusion of terrestrial control groups, sample sizes that satisfy statistical power requirements (i.e., have the ability to detect intervention effects), serial sampling and accounting for individual variability, to optimize personalized risk mitigation. The authors’ review reinforces a call to arms and collective need for a more holistic integrative physiology approach.

In this Viewpoint, I would like to take an opportunity to expand briefly on some of the challenges that the scientific community faces within the context of CM discovery. This begins with a concerted focus on the need to assess an astronaut's (personalized) ‘risk’ profile better and what we, the community, define as ‘acceptable’. This is arguably the biggest ‘ethical elephant’ in the (space) room and a highly emotive topic of ongoing debate, where integrative physiologists have a clear opportunity to contribute.

Current risk tolerances are fortunately far lower than those encountered during the Apollo missions in the 1960s that were by far the most experimental and dangerous, subject to altogether different pressures shaped by the prevailing political and financial climates. Several missions were marred by catastrophe: the Apollo 1 fire killed three astronauts; Apollo 6 was beset by engine shutdown; and the near‐fatal design flaw almost cost the lives of the Apollo 13 astronauts. These events were later compounded by the Space Shuttle disasters (Challenger and Columbia), providing sobering insight into how risky space exploration truly is and that every single precious life ‘counts’. To date, there have been 15 astronaut and 4 cosmonaut fatalities during spaceflight (Schmitz et al., 2022). In the modern era, space agencies have a moral and ethical obligation to provide crews with the best possible estimation of risk to ensure they are adequately informed prior to embarking on space flights, such as a crewed mission to Mars, never before attempted (Antonsen et al., 2023).

As a consequence, there is mounting public and scientific pressure to better characterize and contain the space exposome, to look beyond traditional constraints focused on singular stressors (e.g. microgravity) and to understand the integrative responses of the human body to ‘multi‐stressor’ exposures, including dynamic interplay with genotype (Patel et al., 2020). Decoding the integrated whole, or space ‘integrome’ (not to be confused with space ‘omics’ technologies), although more challenging, has unique potential to unlock unanticipated interactions and combinatorial events that might aggregate to alter allostatic load (cumulative physiological burden) and provide a more realistic estimate of the ‘true’ risk profile of an astronaut (Figure 1a).

This makes intuitive sense, because astronauts invariably encounter exposome stressors to varying degrees and never in isolation. Currently, ‘red’ risks require targeted CM mitigation to ‘downgrade’ them systematically to within ‘acceptable’, albeit currently undefined limits (Figure 1b). Yet current ‘risk boundaries’ are based on single stressors that are (single) organ specific. Our inability to simulate multi‐stressor dynamics accurately and determine to what extent stressors exert (simple) linear additive or (complex) coupled non‐linear synergistic effects (Pirotta et al., 2022) is potentially hampering CM discovery (Figure 1b). Furthermore, and as highlighted by Fernandez‐Gonzalo et al. (2026), different (exercise) CMs impact different organ systems in different ways, with little to no consideration for the integrative dynamics of organ ‘cross‐talk’ and ‘cross‐tolerance’.

A case in point relates to in‐flight application of the advanced resistive exercise device as a CM known to mitigate muscle and bone loss. This can result in an unwelcome elevation in intrathoracic pressure subsequent to intermittent exercise‐induced Valsalva manoeuvres that might intermittently elevate intracranial pressure and corresponding vulnerability to one of the ‘red’ risk neurological complications, spaceflight‐associated neuro‐ocular syndrome (Barisano et al., 2022; Figure 1b). Concurrent integration of an altogether separate (non‐exercise) CM, lower‐body negative pressure, given its ability to reduce intraocular pressure (Greenwald et al., 2021), might prove a complementary CM strategy to optimize systemic exercise benefits (i.e., to eliminate the neurological negatives and to accentuate the postural positives). These inherent complexities clearly argue against a ‘one‐size‐fits‐all’ approach; it is simply too simple!

The downside is that we lack the mechanistic understanding or empirical evidence to derive ‘safe’ or ethically permissible boundary limits for ‘multidimensional’ risks across multi‐organ systems, further emphasizing the fundamental importance of a deeper phenotyping of the space integrome for optimized CM discovery and risk mitigation (Figure 1b). Recent inauguration of LUNA (‘Moon on Earth’) (Casini et al., 2020), Europe's new space analogue facility operated jointly by the European Space Agency and German Aerospace Agency, is an important step in this direction. Specialist input from the wider scientific community has also culminated in White Paper ‘roadmaps’ dedicated to integrative CMs (ESA, 2021) and space governance (Rajput et al., 2023). The latter outlines an ethics framework devoted to fundamental principles and practices to help guide decision‐making by the agency for future exploration‐class missions that are inherently risky and that invariably fail to meet existing (terrestrial occupational) health standards (Rajput et al., 2023). The famous quote of John M. Grunsfeld, American physicist and former NASA veteran of five Space Shuttle flights, adds the all‐important subjective element: ‘All space exploration is risky. As an astronaut, I had to decide each and every time I went to space whether or not to risk my life for the mission. I believe that the future of humans, and the future of Earth, depends on space exploration.’.

Crewed orbital missions to Mars will push physiological tolerances to new and unchartered extremes, exposing crews to complex and poorly characterized risks, which could be uncertain, unforeseen and challenging to manage. Exercise CMs, in combination with others (shielding, nutritional, medicinal and artificial gravity) are key to enabling safe and successful deep space exploration. However, despite astronauts dedicating ∼25% of their precious time aboard the ISS to exercise (Loehr et al., 2015), current CMs remain inadequate and fail to maintain preflight physiological function (Scott et al., 2023). The thought‐provoking topical review by Fernandez‐Gonzalo et al. (2026) makes an important contribution and reinforces the need for further optimization for future exploration missions to the Moon or Mars. Integrative physiologists to the fore!

## AUTHOR CONTRIBUTIONS

Damian M. Bailey conceived the idea and wrote the first draft of the manuscript and revisions thereof. Damian M. Bailey approved the final version submitted for publication and agrees to be accountable for all aspects of the work in ensuring that questions related to the accuracy or integrity of any part of the work are appropriately investigated and resolved. All persons designated as authors qualify for authorship, and all those who qualify for authorship are listed.

## CONFLICT OF INTEREST

D.M.B. is Editor‐in‐Chief of *Experimental Physiology*, past Chair of the Life Sciences Working Group and past member of the Human Spaceflight and Exploration Science Advisory Committee to the European Space Agency. He is a current member of the Space Exploration Advisory Committee to the UK and Swedish National Space Agencies and member of the National Cardiovascular Network for Wales and South‐East Wales Vascular Network. D.M.B. is also affiliated to Bexorg, Inc. (USA) focused on the technological development of novel biomarkers of cerebral bioenergetic function and structural damage in humans.
